# Debulking Atherectomy in the Peripheral Arteries: Is There a Role and What is the Evidence?

**DOI:** 10.1007/s00270-017-1649-6

**Published:** 2017-04-27

**Authors:** Konstantinos Katsanos, Stavros Spiliopoulos, Lazaros Reppas, Dimitris Karnabatidis

**Affiliations:** 1 Department of Interventional Radiology, School of Medicine, Patras University Hospital, Panepistimiou St., 26504 Rion, Greece; 2 Interventional Radiology Unit, 2nd Department of Radiology, ATTIKO Athens University Hospital, 1st Rimini St., Chaidari, 12461 Athens, Greece; 3 Guy’s and St.Thomas’ NHS Foundation Trust, London, United Kingdom

**Keywords:** Atherosclerosis, Plaque excision, Atherectomy, Rotational, Directional, Laser, Embolization, Patency, Amputation

## Abstract

Traditional percutaneous balloon angioplasty and stent placement is based on mechanical plaque disruption and displacement within the arterial wall. On the contrary, transcatheter atherectomy achieves atherosclerotic plaque clearance by means of directional plaque excision or rotational plaque removal or laser plaque ablation. Debulking atherectomy may allow for a more uniform angioplasty result at lower pressures with consequently less vessel barotrauma and improved luminal gain, thereby decreasing the risk of plaque recoil and dissection that may require permanent metal stenting. It has been also argued that atherectomy may disrupt the calcium barrier and optimize drug transfer and delivery in case of drug-coated balloon applications. The authors discuss the various types of atherectomy devices available in clinical practice to date and critically appraise their mode of action as well as relevant published data in each case. Overall, amassed randomized and observational evidence indicates that percutaneous atherectomy of the femoropopliteal and infrapopliteal arteries may achieve high technical success rates and seems to lessen the frequency of bailout stenting, however, at the expense of increased risk of peri-procedural distal embolization. Long-term clinical outcomes reported to date do not support the superiority of percutaneous atherectomy over traditional balloon angioplasty and stent placement in terms of vessel patency or limb salvage. The combination of debulking atherectomy and drug-coated balloons has shown promise in early studies, especially in the treatment of more complex lesions. Unanswered questions and future perspectives of this continuously evolving endovascular technology as part of a broader treatment algorithm are discussed.

## Introduction

Peripheral artery disease (PAD) is an occlusive pathology of the extremities—mainly lower—affecting over 200 million people worldwide, while in the past decade, its prevalence increased by 28.7 and 13.1%, in low- to mid-income and high-income countries, respectively [1]. Arteriosclerosis with atheroma formation is the basis of PAD pathophysiology, leading to tissue hypo-perfusion and gradual progression until complete occlusion, with increasing degrees of ischemia. Arterial lumen stenosis occurs due to gradual atheroma formation within the tunica intima following accumulation of mostly macrophage cells, cholesterol, fatty acids, calcium and fibrous connective tissue [2]. Arteriosclerosis is a chronic systemic inflammatory disorder, leading to both chronic and acute multi-vascular lesions due to atheromatous plaque development presenting with symptomatic vessel stenosis and occlusion, or even plaque rupture and thrombosis. Development of PAD is often correlated with the more aggressive forms of the disease, which may include a wide concomitant vascular involvement, including but not limited to calcific coronary artery disease, impaired vessel remodeling, progression to critical limb ischemia and poor cardiovascular prognosis [3].

The mechanism of endovascular treatment using balloon angioplasty and stenting is based on plaque disruption and displacement within the arterial wall [4]. Consequently, the atheroma is not removed but pressed or crushed by the balloon and redistributed inside and along the arterial wall. As a result, in cases of hard, eccentric, severely calcified, atherosclerotic disease, balloon angioplasty performs poorly and vessel recoil is frequent, while simply “caging” the atheromatous plaque using a stent also may also lead to suboptimal immediate result and/or rapid relapse of the steno-occlusive lesion [5]. Moreover, stent placement is not advisable in certain anatomical locations such as the distal foot arterial system, while flexion points such as the hip and knee joints could provoke stent deformation or fracture leading to arterial occlusion. Moreover, permanent deployment of a metallic mesh at the arterial wall has been implicated with in-stent restenosis, total occlusion or thrombosis which may cause severe difficulties in retreating the lesion percutaneously [6].

Surgical endarterectomy is considered the gold standard of common femoral and carotid artery atherosclerotic disease, as it offers complete atherosclerotic plaque removal combined with very satisfactory patency rates [7]. Endovascular atherectomy has emerged as a novel, endovascular technology for atheroma removal, offering the benefits of both surgical endarterectomy and minimally invasive, percutaneous treatment. Percutaneous atherectomy could therefore present an effective alternative for the treatment of chronic total occlusions and eccentric, fibro-calcific, plaques and Mönckeberg’s medial calcinosis characteristic in diabetic patients, which respond poorly to angioplasty or stenting or both. The debulking effects of its mechanism of action may theoretically allow for a more uniform angioplasty result with minimal consequent vessel barotrauma and improved luminal gain, thereby decreasing the risk of plaque recoil and dissection, and preventing negative remodeling and neointimal hyperplasia [8]. In this review, authors will present all endovascular atherectomy devices available in clinical practice to date and will critically appraise their mode of action as well as relevant published data. Unanswered scientific questions and future perspectives of this interesting emerging endovascular technology will also be discussed.

## General Considerations

Endovascular atherectomy may be performed under local anesthesia using standard caliber arterial sheaths, ranging from 4 to 8Fr, and provides the theoretical advantage over balloon angioplasty that plaque is removed rather than pressed against the arterial wall, and subsequent balloon dilation is optional depending on the debulking effect. This contributes to substantial luminal gain with less barotrauma even if post-dilation is performed, decreasing the risk of dissection and/or neointimal hyperplasia, while avoiding stent placement. The latter omits the permanent inflammatory stimulus from the metallic stent mesh and facilitates future re-interventions including bypass surgery.

Currently, the only relative contraindications to endovascular atherectomy for infrainguinal lesions are subintimal lesion crossing and minimum vessel diameter smaller than that indicated for each device according to the instructions for use (IFU). Longer procedural time, larger sheath diameters, lesion (vessel diameter, long occlusions, stenosis, in-stent restenosis) and patient characteristics (comorbidities, clinical presentation, performance status, etc.) should be cautiously assessed and considered on a case-by-case basis.

Endovascular atherectomy devices can be divided into four categories according to the mechanism used for atheroma removal: directional, rotational or orbital and laser atherectomy devices. There are also chronic total occlusion (CTO) recanalization devices also approved for atherectomy. Until today, there are no data regarding the comparison of different atherectomy devices in PAD patients, while each device presents unique features with discrete advantages and disadvantages. All available devices and their technical characteristics are presented in Table 1, while possible advantages and disadvantages of each atherectomy category are outlined in Table 2. Purely thrombectomy devices without any plaque removal capacity (AngioJet, Penumbra, etc.) are not discussed in the present manuscript.

**Table 1:** Peripheral atherectomy devices categorized according to the type of atherectomy performed and their basic technical characteristics

	Technical characteristics
*Directional atherectomy*	
SilverHawk™ (Medtronic, MN, USA)	Side-cutting single rotating blade, collecting nosecone, no active aspiration
TurboHawk™ (Medtronic, MN, USA)	Side-cutting four contoured blades, collecting nosecone, no active aspiration
HawkOne™ (Medtronic, MN, USA)	Side-cutting single rotating blade, preloaded distal flush tool, collecting nosecone, no active aspiration
Pantheris (Avinger Inc., CA, USA)	OCT-guided atherectomy, side cutter, apposition balloon, collecting conenose no active aspiration
*Rotational atherectomy*	
Pathway Jetstream PV (Boston Scientific, MN, USA)	SC catheter: front cutting blades. XC catheter: second set of larger blades. Acute thrombus and atheroma removal, active debris aspiration
Peripheral Rotablator™ (Boston Scientific MN, USA)	Diamond-coated burr, luminal gain matches the size of the burr, no active aspiration
Phoenix (AtheroMed Inc., CA, USA)	Front cutter, mechanical (active) debris removal
Rotarex^®^ S (Straub Medical, Wangs, Switzerland)	Thrombectomy/atherectomy device, external metallic rotating tip and internal helix with aspiration function
*Orbital atherectomy*	
Diamondback 360° (Cardiovascular Systems Inc., MN, USA)	Eccentric diamond-coated crown, atherectomy depth increasing with speed, no active aspiration
*Excimer laser atherectomy*	
Turbo-Tandem, Turbo-Elite and Turbo-Power catheters (Spectranetics Corporation, CO, USA)	Ultraviolet radiation to remove atheroma. FDA for in-stent restenosis and de novo lesions. Turbo-Elite: occlusion crossing without guide wire. No active aspiration
*CTO/atherectomy devices*	
Crosser peripheral CTO recanalization system (Bard Peripheral Vascular Inc., AZ, USA)	High-frequency mechanical vibrations, transmitted to a metallic tip. Saline flush cooling system. Over-the-wire and rapid exchange. No active aspiration

**Table 2:** Reported advantages and disadvantages of different atherectomy types

Type	Advantages	Disadvantages^a^
Directional atherectomy	Targeted eccentric plaque removal, effective in severely calcified lesions	Vessel wall trauma, time-consuming (multiple passes, discharge debris from conenose)
OCT-guided directional atherectomy	Image-guided targeted plaque removal avoiding normal vessel wall	Time-consuming (multiple passes, discharge debris from conenose)
Rotational atherectomy	Effective in severely calcified lesions, active aspiration, very fast	Cannot moderate the depth of atherectomy
Orbital atherectomy	Effective in severely calcified lesions, atherectomy range modified with speed (one cone for multiple vessels)	Not effective for ISR
Laser excimer atherectomy	FDA for ISR. Effective in severe calcifications	Time-consuming (slow pass rate to deliver energy)
CTO devices	Facilitates CTO crossing	Suboptimal luminal gain

^a^Major disadvantages for all atherectomy devices are the possibility of distal embolization, contraindication following subintimal lesion crossing and increased overall procedural time

## Directional Atherectomy

In directional atherectomy, plaque is removed by guiding the cutting device (cutter) of the catheter directly to the plaque, while by rotating the catheter to the preferred direction, the device accomplishes targeted atherosclerotic plaque removal. This is an advantage when treating eccentric lesions. Reasonable plaque volume is removed only by multiple passes. Removed plaque is packed into the nosecone, and after few passes, the catheter must be retrieved and the nosecone must be emptied in order to proceed with further debulking.

The SilverHawk™, TurboHawk™ and the newest HawkOne™ directional atherectomy, plaque excision systems (Medtronic, MN, USA) have received approval from the US Food and Drug Administration (FDA) for use in peripheral arterial lesion. The SilverHawk™ is a side-cutting cutting device. The catheter is equipped with a single rotating blade within a tubular cover which ends at a nosecone (collection area). The rotating blade is powered by a motor within the capital equipment. The TurboHawk™ is a similar system with four contoured blades, achieving more plaque removal with each pass and provides more aggressive atherectomy which is an advantage in the treatment of severely calcified lesions. The HawkOne™ is the most recent directional atherectomy catheter of this series and has been designed to provide more effective treatment for calcified lesions. It is a one blade, 7Fr platform with lower crossing profile, equipped with a preloaded distal flush tool simplifying the cleaning process necessitating less procedural steps (55% less time) and providing two times more cutting efficiency than the TurboHawk™. Although these devices do not have an aspirating mechanism, the majority of excised plaque is collected within the nosecone. Once the nosecone is full, the device must be retrieved and emptied before further use. Nevertheless, distal embolization remains an issue and their use without an appropriate protection arterial filter is not advisable. All three devices are available in various sizes for use in vessels with diameters ranging from 1.5 to 7 mm [9].

A novel directional atherectomy device which recently received FDA clearance is the Pantheris OCT-guided lumivascular atherectomy device (Avinger Inc., CA, USA). The Pantheris over-the-wire catheter is equipped with optical coherence tomography (OCT) technology to enhance directional atherectomy efficacy and safety, allowing targeted removal of eccentric plaque (characteristic of directional atherectomy), while minimizing the risk of non-diseased vessel wall trauma [10]. The catheter is using a side cutter with a conenose similar to the previously described directional atherectomy devices, without aspiration capability, but also utilizes an apposition balloon which enables OCT-guided depth modification of atherectomy. Moreover, direct visualization of the arterial lumen during atherectomy, without using ionizing radiation, reduces procedural X-ray exposure. The system is compatible with 7 or 8Fr sheaths (2 different catheters) and is indicated for the treatment of vessels ranging from 3 to 7 mm in diameter, but is not recommended for treating iliac, renal or carotid artery lesions [10].

## Rotational Atherectomy

**Fig. 1:**
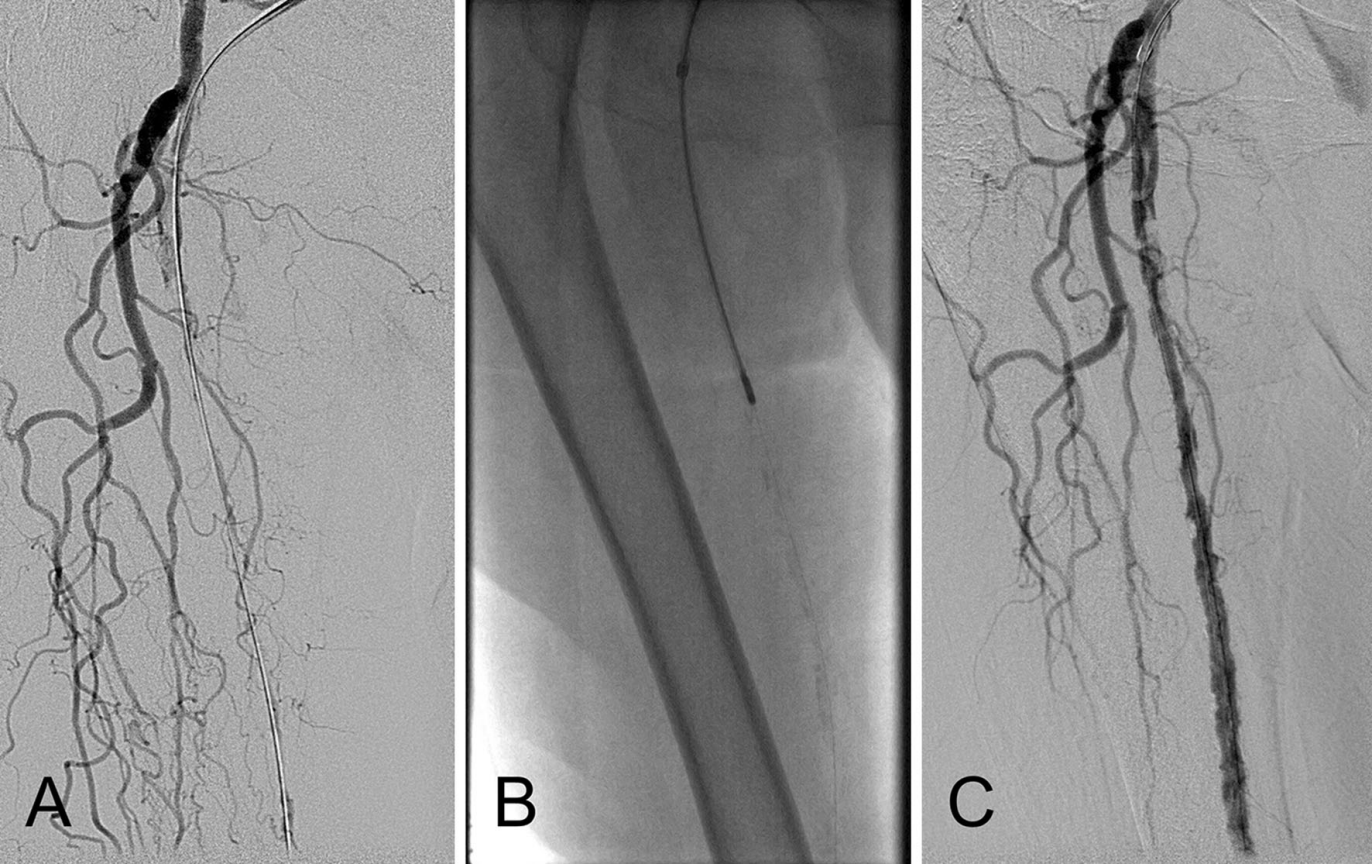
Example of plaque debulking. **A** Baseline image of long-segment total occlusion of the right superficial femoral artery in a female patient with critical limb ischemia. **B** Rotational–aspiration atherectomy with the JETSTREAM 2.4/3.4 device. **C** Immediate post-atherectomy result after two passes—one with the blades down and the second with the blades up. Note that no balloon has been used yet

In rotational atherectomy, plaque is excised by a concentrically rotating, specially designed tip (burr). As a result, luminal gain usually matches the size of the tip/burr used, and if a larger lumen is necessary, a larger catheter tip/burr should be utilized. The Pathway Jetstream PV Atherectomy System (Boston Scientific, MN, USA) is a cutting rotational atherectomy device with active debris aspiration, indicated for both acute thrombus removal and atherectomy of chronic lesions. It is a 7Fr sheath, over-the-wire system with two types of catheters: the SC catheter which is smaller, equipped with a single set of front cutting blades and the larger XC catheter equipped with a second set of larger blades proximal to the front cutting set that can be used in order to increase the diameter of the debulking effect of the atherectomy (Fig. 1). The aspiration port is situated proximal to these larger blades, and even more proximal infusion ports are enabling flushing during use. The Jetstream console (capital equipment) is designed to enable atherectomy, active aspiration, flushing and monitoring of the volume of blood products removed. Despite active aspiration, micro- and macro-embolization is possible, so filter protection is again advisable.

The Peripheral Rotablator™ system (Boston Scientific MN, USA) consists of an outside console that rotates concentrically a 5-micron diamond-coated catheter tip (burr). The unique feature of this device is that the luminal gain is predefined as it matches the size of the burr (ranges from 1.25 to 2.5 mm) creating a smooth lumen with specific diameter. However, as a 1.5-mm-diameter guide is integrated to the catheter, narrow lesions may be difficult to cross. The catheter requires 4 to 8Fr arterial sheaths and is compatible with a specific 0.009′′ guide wire. The maximum atherectomy time recommended for a single catheter is 15 min; after that, the burr is considered ineffective. Active aspiration is not available. The diamond-coated burr macerates the atheroma in debris smaller than red blood cells, which usually do not incite clinically significant embolization. Nevertheless, filter protection is recommended.

The Phoenix rotational atherectomy system (AtheroMed Inc., Menlo Park, CA, USA) consists of two main components: a single-use catheter without capital equipment and the Phoenix atherectomy handle. The long, flexible double-lumen catheter contains a torque shaft attached to a metallic front cutter at its distal tip. The rotating torque shaft enables rotational atherectomy by the cutter. The excised plaque is mechanically transported within the catheter using an Archimedes screw fixed on the outer surface of the shaft extended though the entire length of the catheter with a port on the handle connected with an external bag. The device received FDA clearance for peripheral use recently and is available in sizes ranging from 1.8 to 2.4 mm, compatible with 0.014 inch, 260 cm length guide wire and 5 to 7Fr sheaths. According to the IFU, there is no plaque volume limit that can be excised, and the minimum vessel diameter recommended for treatment is 3 mm. Again micro- and macro-embolization is possible and filter protection is recommended.

Finally, the Rotarex^®^ S (Straub Medical, Wangs, Switzerland) is a rotational thrombectomy device that that can be also used as an atherectomy device for chronic total occlusions. It has an active aspiration function enabling debris removal within an external bag. As the external metallic device rotates, the internal metallic helix rotates at a speed of 40.000–60.000 rounds per minute, creating a negative pressure and removing debris from circulation. It is compatible with sheaths from 6 to 10Fr and can be used to treat vessels from 3 to 8 mm in diameter. It may be advisable to combine use of thrombectomy devices like the Rotarex with peripheral filter protection to reduce the risk of distal thromboembolism [11].

## Orbital Atherectomy

Orbital atherectomy is a new endovascular atherectomy mechanism based on the high-speed rotational spin of the shaft and the orbital rotation of a specially designed debulking, diamond-coated crown. Plaque is removed by the orbital movement of the crown, while the debulking area increases with the increase of the rotational speed of the crown. This is the main difference compared to rotational atherectomy which uses a concentrically rotating burr, so luminal gain is as large as the burr size being utilized and is not modified by increased rotational speed.

It is currently performed only with the Diamondback 360° Peripheral Orbital Atherectomy System (Cardiovascular Systems Inc., St. Paul, MN, USA), which consisted of an orbiting eccentric diamond-coated crown mounted at the end of a shaft and a capital equipment (OAS pump). It is a 0.014-inch over-the-wire system using a proprietary guide wire (the ViperWire™), which provides more crossing support to that of a 0.009-inch guide wire used in rotational atherectomy, and it is the only atherectomy system compatible with 4Fr sheaths (up to 7Fr). Three types of crowns are available: A solid micro-crown is recommended for tortuous vessel anatomy, tight bends and distal below the ankle lesions, a solid crown is recommended for calcified lesions and maximum plaque removal in the short atherectomy time (additional diamond-coated surface area), and the classic crown is the most flexible and recommended for below the knee lesions. There is no aspiration function, and although small particulates created from crown rotation are not considered particularly hazardous, distal embolization cannot be excluded and the use of a peripheral protection filter is advised.

## Laser Atherectomy

Laser atherectomy uses excimer laser technology to ablate atheromatous peripheral arterial disease. Excimer laser atherectomy catheters (Turbo-Elite, Turbo-Power and Turbo-Tandem Spectranetics Corporation, Colorado Springs, CO, USA) are using ultraviolet radiation to remove atheroma from the arterial lumen with a thickness of 10 μm with each pulse of energy [9]. Excimer laser technology utilizes low penetration depth and pulsed delivery of high energy as to achieve the disruption of the atheroma that is in contact with the laser without damaging the surrounding arterial tissue. Laser atherectomy is indicated for both de novo and in-stent restenosis. The Turbo-Tandem is not designed to be used in total or subtotal occlusions, while the Turbo-Elite catheter is capable of crossing occlusions without the need of a guide wire. Catheter diameters range from 0.9 to 2.5 mm and are compatible with 4–8Fr sheaths. The device is powered by an external generator (CVX-300 excimer laser ablation system) and is most effective in a ratio of 2:3 with respect to catheter/vessel diameter. Catheter advancement is also important in delivering the appropriate amount of energy to the lesion and should be performed slowly with a rate of ≥0.5 ≤ 1 mm/s so as to remove plaque effectively and uniformly [12]. Importantly, laser should never be used in the presence of contrast media as this increases energy absorption leading to dissection or perforation. As blood also absorbs laser energy, saline flushing during laser atherectomy is essential in order to remove blood and contrast from the treated vessel. Micro- and macro-embolization has been described and the use of a protection filter is advisable.

## CTO Systems with Atherectomy Capabilities

The Crosser peripheral CTO recanalization system (Bard Peripheral Vascular Inc., Tempe, AZ, USA) has been developed as a CTO crossing system. However, during CTO, crossing operators noticed that after crossing the occlusion the device was creating a considerable patent tract corresponding to the catheter’s outer diameter or even larger and was therefore granted with FDA clearance for atherectomy. The system consists of capital equipment (generator and transducer) and a single-use disposable catheter. The capital equipment converts alternative current into high-frequency mechanical vibrations, transmitted through the catheter’s nitinol wire to its metallic tip. Saline flush is used to cool the tip of the catheter while in use. The system is compatible with sheaths and is available in over-the-wire and rapid exchange versions. The device is effective in crossing hard, calcified occlusions, but due to its specific CTO, crossing mechanism of action balloon angioplasty is required in the majority of the cases.

## Randomized Controlled Trials (RCT)

In the past few years, both femoropopliteal and infrapopliteal percutaneous atherectomy were investigated in several multicenter, randomized controlled trials (RCT), as well as in large-scale multicenter registries and retrospective analysis. In 2011, Shammas et al. published outcomes from a two-center, RCT comparing primary balloon angioplasty versus SilverHawk directional atherectomy with adjunctive balloon angioplasty in 58 patients suffering from intermittent claudication (IC; 46 patients) or critical limb ischemia (CLI; 12 patients). The majority of the cases (46/58 patients; 79%) involved femoropopliteal lesions, while in the remaining 21%, infrapopliteal lesions were also treated [13]. Procedural variables such as lesion length, stenosis severity, total occlusions and extent of vessel calcification were similar between the two study arms. The primary endpoint of the study was set at target lesion revascularization (TLR) at 1 year, while secondary endpoints included technical success, bailout stenting rate and target vessel revascularization (TVR). Technical success was 100% in the angioplasty arm versus 97.2% in the atherectomy arm. During follow-up, TLR (11.1 vs. 16.7%) and TVR (11.1 vs. 21.4%) were all similar in the atherectomy and angioplasty arms, respectively, but with a small numerical benefit in favor of directional atherectomy. However, atherectomy plus angioplasty resulted in significantly less bailout stenting due to suboptimal immediate technical result (27.6 vs. 62.1%; *p* = 0.017). On the other hand, distal macro-embolization was significantly higher in the atherectomy arm (64.7 vs. 0.0%; *p* < 0.001) [13].

The same research group published later the results from another multicenter, RCT investigating infrapopliteal orbital atherectomy with adjunctive balloon angioplasty versus balloon angioplasty alone in a total of 50 patients presenting with CLI (Rutherford class 4 to 6) due to severe calcified infrapopliteal lesions [14]. Procedural success (atherectomy plus angioplasty 93.1% vs. angioplasty alone 82.4%; *p* = 0.27) and stent use (atherectomy plus angioplasty 6.9% vs. angioplasty alone 14.3%; *p* = 0.44) were similar between the two groups. At 1-year follow-up, no patient underwent major amputation, while freedom from target vessel revascularization and all-cause mortality rates were 93.3 and 100% in the atherectomy plus balloon angioplasty arm versus 80.0% (*p* = 0.14) and 68.4% (*p* = 0.01) in the balloon angioplasty alone arm, respectively. Post hoc analysis detected a 5.6 hazard ratio for major adverse events in cases of acute post-procedure residual stenosis >30% (*p* = 0.01). Based on these results, the authors concluded that orbital atherectomy could increase the probability of achieving an optimal angioplasty outcome and lead to fewer dissections, decreased bailout stenting rate and statistically significantly lower adjunctive balloon pressure compared to balloon angioplasty alone [14].

Another multicenter RCT, published in 2014, report immediate and midterm outcomes of orbital atherectomy plus balloon angioplasty compared to balloon angioplasty alone, in 50 patients with 65 calcified femoropopliteal lesions. Study’s primary endpoint was freedom from TLR (including adjunctive stenting), or Duplex ultrasound (DUS) defined restenosis at 6 months [15]. Stent was deemed necessary in 5.3% in the atherectomy arm and in 77.8% in the balloon angioplasty arm (*p* < 0.001). Freedom from TLR including adjunctive stenting or restenosis was noted in 77.1 versus 11.5% (*p* < 0.001) at 6 months and in 81.2 versus 78.3% at 1 year, excluding adjunctive stenting (*p* > 0.99), in the atherectomy and balloon angioplasty arm, respectively. Again, although less stent use was noted, atherectomy did not yield superior outcomes compared to standard balloon angioplasty [15].

The possible benefit from transcatheter debulking in the treatment of challenging femoropopliteal in-stent restenosis was investigated in a multicenter RCT by Dippel et al. In total, 250 patients presenting with IC or CLI (Rutherford Class 1 to 4) due to ISR were randomized (2:1 ratio) to undergo excimer laser atherectomy (ELA) with plain balloon angioplasty versus balloon angioplasty alone, in 40 US centers [16]. Primary efficacy endpoint was 6-month TLR and primary safety endpoint was 30-day major adverse event (death, amputation or TLR). Mean lesion length was similar around 19 cm, and in over 30% of the cases, total occlusions were treated, while the rate of vessels presenting calcifications was significantly higher in the ELA arm (27.1 vs. 9.1%; *p* = 0.002). ELA resulted in superior procedural success (93.5 vs. 82.7%; *p* = 0.01) and freedom from TLR (73.5 vs. 51.8%; *p* < 0.005), significantly fewer procedural complications and 30-day major adverse event rates (5.8 vs. 20.5%; *p* < 0.001), respectively. Moreover, ELA was associated with a 52% TLR reduction. The authors concluded that ELA plus balloon angioplasty significantly improves acute and midterm efficacy and safety outcomes of femoropopliteal ISR treatment compared with conventional PTA alone [16].

Finally, a 2014 meta-analysis summarized the outcomes of percutaneous transcatheter atherectomy in the femoropopliteal segment. The evidence synthesis included six RCTs comprising 287 patients (328 lesions) treated with atherectomy or balloon angioplasty for femoropopliteal artery disease alone [17]. Technical success, bailout stenting and distal arterial embolization were similar between the atherectomy and the angioplasty group. The 9-month primary patency was also similar between the two groups (Risk ratio: 0.90, 95% CI 0.56–1.46, *p* = 0.68, *I* ^2^ = 69%). The authors concluded that these results did not show any procedural advantage or clinical improvement following debulking atherectomy of the femoropopliteal artery compared to plain balloon angioplasty alone. Nonetheless, this meta-analysis was based on limited, low-quality, heterogeneous evidence with high risk of bias [17].

## Multicenter Prospective Registries and Large Retrospective Cohorts

### Directional Atherectomy

Among the first large prospective multicenter registries for peripheral endovascular atherectomy was the TALON study which involved 19 US institutions as to investigate directional atherectomy with the SilverHawk device in 601 patients (748 limbs) with IC or CLI due to femoropopliteal and/or infrapopliteal disease. Mean lesion lengths were 62.5 mm for femoropopliteal lesions and 68.5 mm for infrapopliteal lesions [18]. Procedural success was 97.6%. Atherectomy alone without adjunctive treatment was performed in 73.3% of the lesions, while stent deployment was necessary in 6.3% of the lesions. The 6- and 12-month rates of freedom from TLR were 90 and 80% at 6- and 12-month follow-up, respectively. According to multivariate analysis, history of MI or coronary revascularization (HR 5.49, 95% CI 1.87–16.10), multiple lesions (HR 1.37, 95% CI 1.11–1.70) and increasing Rutherford category (HR 1.84, 95% CI 1.28–2.65) were significant predictors of 6-month TLR. Lesion length > 50 mm was associated with a 2.9-fold increased risk of TLR (HR 2.88, 95% CI 1.18–7.01); lesion length > 100 mm was associated with a 3.3-fold increase in TLR (HR 3.32, 95% CI 1.15–9.56) [18].

In 2014, data from the DEFINITIVE LE (Determination of EFfectiveness of the SilverHawk PerIpheral Plaque ExcisioN System (SIlverHawk Device) for the Treatment of Infrainguinal Vessels/Lower Extremities), were published. This was the largest prospective, multicentered, real-world registry, conducted in 47 multinational centers to investigate directional atherectomy for infrainguinal lesions up to 20 cm in a total of 800 patients. Primary endpoints were primary patency at 1 year by DUS assessed by independent core laboratory analysis for claudicants and freedom from major unplanned amputation decided by a clinical events committee for patients suffering from CLI, while the study was powered for a non-inferiority assessment of primary patency in diabetic versus nondiabetic claudicants. Primary patency at 1 year was 78% and was similar between the diabetic subgroup and nondiabetic subgroups (77 vs. 78%; *p* < 0.001). Freedom from major unplanned amputation rate was 95%. Peri-procedural adverse events were embolization (3.8%), perforation (5.3%) and abrupt vessel occlusion (2.0%), while bailout stent rate was 3.2%, significantly less to that reported by Shammas et al. [13]. Nearly 40% of the lesions were calcified, and in approximately 21% of the cases, occlusions were treated. According to an indirect comparison with published data, the authors claimed that atherectomy provided similar patency outcomes with various stent and drug-coated balloon trials such as RESILIENT, STRIDES, LEVANT I, DURABILITY II and the Zilver RCT randomized study [19, 20].

Of further interest, recently, the DEFINITIVE LE Investigators reported outcomes of the infrapopliteal atherectomy subgroup which included 145 subjects with 189 infrapopliteal lesions (48.3% CLI and 68.3% diabetic patients). Mean lesion length was 58 ± 44 mm and 20.2% were occlusions. Primary patency was 84% at 1-year follow-up (89.6% for claudicants and 78% for CLI; *p* = 0.11), while overall freedom from major amputation was 97.1% (100% in claudicants and 93.8% in CLI; *p* = 0.03). Significant clinical improvements and quality-of-life measurements were noted at 1 year in both claudicants and CLI subgroups [21]. The DEFINITIVE-Ca ++ investigators looked further into the outcomes of directional atherectomy under filter protection for the treatment of moderately to heavily calcified femoropopliteal lesions in particular. The analysis included 133 patients with 168 lesions, and the primary effectiveness endpoint (defined as <50% residual stenosis) was achieved in 92% of the cases [22].

Zeller et al. reported long-term results of a prospective single-center registry investigating SilverHawk directional atherectomy in femoropopliteal lesions. In total, 84 patients (100 limbs) with Rutherford 2 to 5 disease were included. Technical success rate was 86% for atherectomy only, but reached up to 100% after additional low-pressure balloon angioplasty (59%) or stenting (6%). Primary patency according to Duplex was 84% in de novo lesions, 54% for native vessel and 54% for in-stent restenosis at 12 months (*p* = 0.002) and 73, 42 and 49%, at 18 months, respectively (*p* = 0.008). In total, six distal embolization events occurred (6%) [23].

### Rotational Atherectomy

**Fig. 2:**
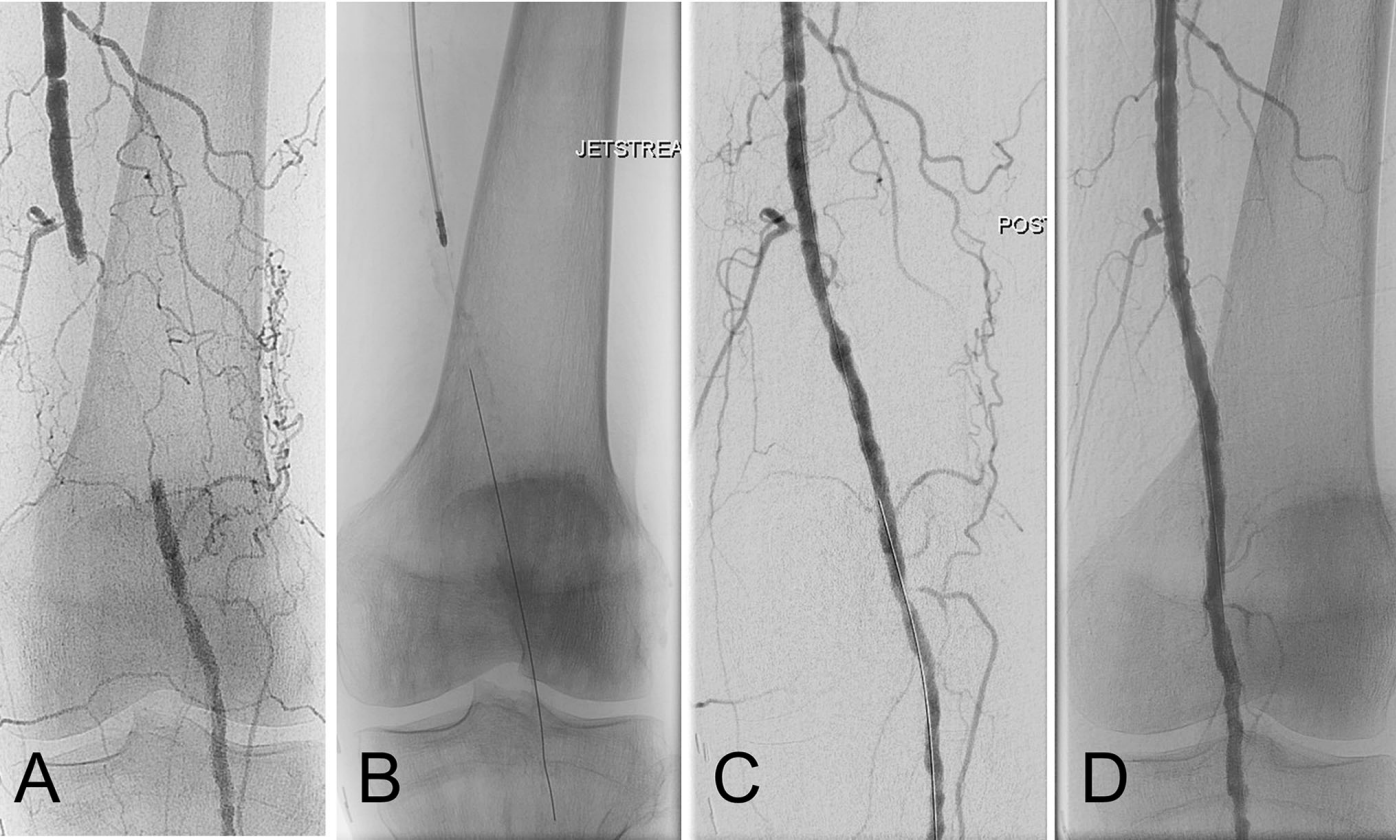
Percutaneous popliteal atherectomy. **A** Baseline antegrade angiogram of a 5-cm chronic total occlusion of the proximal left popliteal artery (P1 segment) in a young male claudicant patient. **B** Rotational–aspiration atherectomy with the JETSTREAM 2.4/3.4 device. **C** Immediate post-atherectomy result after two passes (blades down and blades up) shows a very good atherectomy result with minimal residual stenosis. **D** Completion angiogram after adjunctive paclitaxel-coated balloon angioplasty to inhibit late restenosis. The vessel was found to be widely patent at DUS at 1-year follow-up

**Fig. 3:**
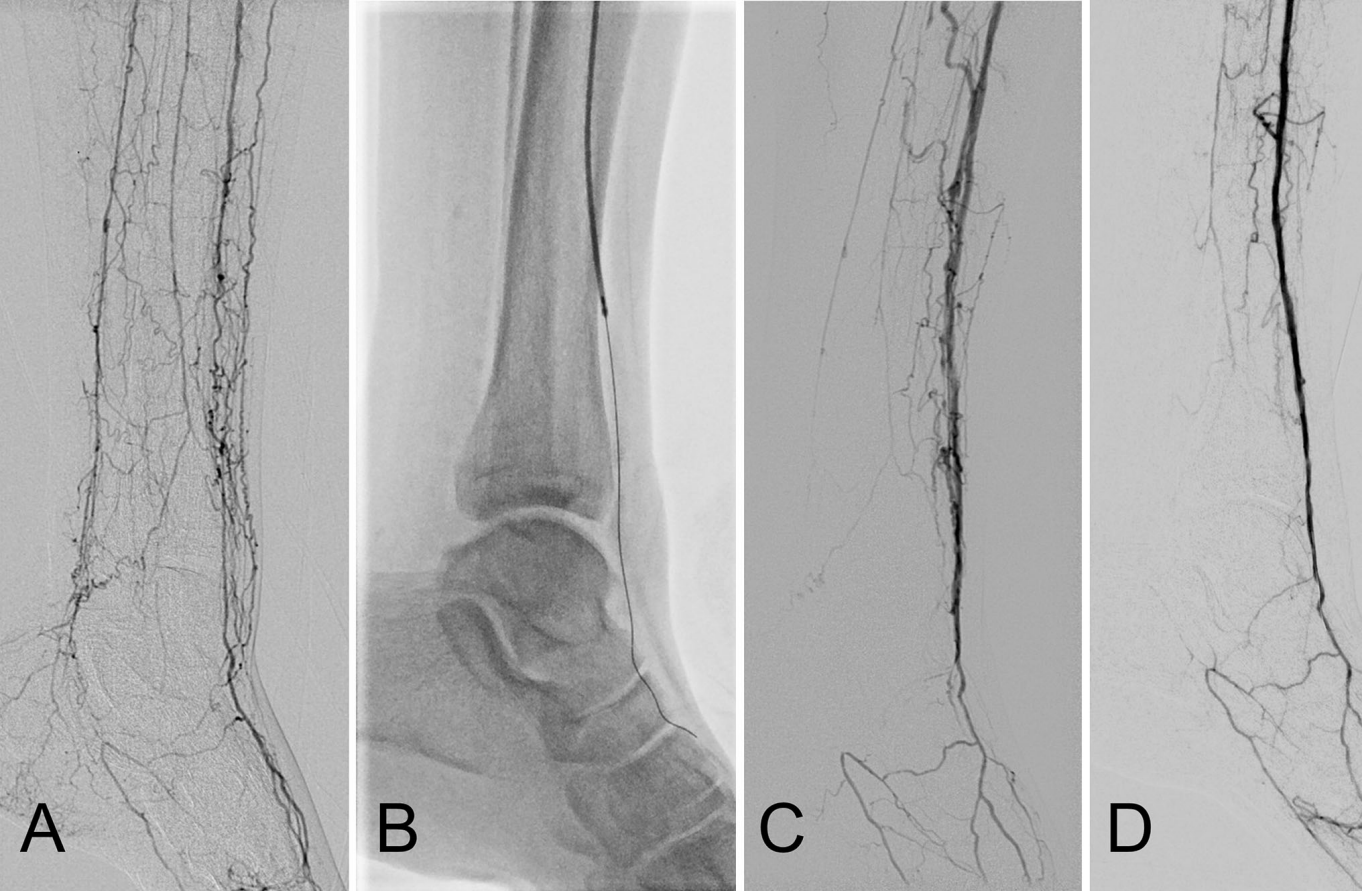
Infrapopliteal debulking atherectomy. **A** Elderly male patient with an ischemic previously debrided left hallux wound. Baseline subtraction angiography demonstrates long-segment occlusion of the posterior tibial artery and segmental occlusion of the distal third of the anterior tibial artery with reconstitution of the dorsalis pedis through collateral networks. **B** Antegrade rotational atherectomy with the PHOENIX device following a complex subintimal–intraluminal recanalization that required a combined pedal puncture. **C** Immediate post-atherectomy result after several passes shows a good atherectomy result with some early venous filling. **D** Completion angiogram after adjunctive 3-mm-long balloon angioplasty demonstrates a very good anatomical result with brisk antegrade filling of the pedal circulation. The wound healed successfully 3 months later

The Pathway PVD Trial is a large multicenter, prospective registry that investigated rotational atherectomy with aspiration in both femoropopliteal and infrapopliteal lesions (Figs. 2, 3). The study included 172 patients treated for either IC or CLI in nine European cites. In total, 31% of the cases occlusions were treated, while 51% of the lesions presented moderate to high calcium score. Device success was 99% and only two preplanned amputations were noted. Clinically driven TLR rates at 6 and 12 months were 15 and 26%, respectively, while 1-year DUS-detected restenosis rate was 38.2%. Both ankle-brachial index and mean Rutherford class significantly increased at 12 months [24].

Rotational atherectomy and aspiration atherectomy (Pathway system) were also investigated in 33 consecutive patients (40 lesions; mean lesion length 85.7 mm) with infrainguinal ISR in a European multicenter, prospective registry involving five cites. In 57.5% of the cases, adjunctive additional plain balloon angioplasty was performed. Although ABI and Rutherford class significantly improved compared to baseline and no major device-related adverse events were noted, 1-year angiographic outcomes were disappointing as primary patency was only 33 and 25% at 12- and 24-month follow-up, respectively [25].

Just recently, Mehta et al. published their outcomes from a retrospective study investigating common femoral artery rotational JETSTREAM atherectomy with adjunctive balloon angioplasty and provisional stenting versus plain balloon angioplasty. Data were obtained from a prospectively maintained database, and 167 patients with Rutherford 3 to 4 disease were included in the analysis. Mean follow-up was 42.5 months. Major adverse events (major bleeding, pseudoaneurysm, thrombosis and distal embolization) were 3.0%. Patients in the PTA-only group had a significantly lower patency compared with the atherectomy plus PTA group. In addition, the CFA provisional stent group demonstrated a surprising 100% primary patency rate, significantly superior to that achieved from non-stent groups (77.0%; *p* = 0.0424) [26].

The OASIS investigators reported the immediate and early clinical outcomes following application of orbital atherectomy in 201 chronic infrapopliteal stenoses of 124 patients as part of an FDA IDE investigation. The primary safety endpoint of major adverse events (MAE) at 30 days occurred in four patients (3.2%), whereas the primary post-treatment diameter stenosis was 17.8 ± 13.5%. At 6 months, MAE was observed in 10.4% of the cases, no patients required surgical bypass or unplanned amputation, and an improvement in the Rutherford ordinal scale was observed in 78.2% of patients [27].

### Laser Atherectomy

In the Laser Angioplasty for Critical Limb Ischemia (LACI) prospective registry, which was performed at 14 sites in the USA and Germany, 145 patients (155 limbs) with femoropopliteal or BTK disease underwent excimer laser-assisted endovascular treatment. Mean treatment length was over 16 cm and occlusions were treated in 92% of limbs. The 6-month limb salvage rate was 93%, while stents were implanted in 45% of the cases [28]. In another study by Scheinert et al., 411 SFA long-segment occlusions were recanalized with laser-assisted angioplasty with an average lesion length of 19.4 cm achieving technical success of 90.5%. Complications included acute re-occlusion (1%), perforation (2.2%) and distal embolization (3.9%), while the 1-year assisted primary and secondary patency rates were 65.1 and 75.9%, respectively [29]. Interestingly, in contrary to excisional atherectomy options, the risk of distal embolization with laser use in the lower extremity was found to be comparable to the risk after angioplasty and stenting [30].

The TURBO-Booster catheter (Spectranetics) was studied in the multicenter clinical trial ClirPath Excimer Laser to Enlarge Lumen Openings (CELLO), and results showed a high procedural success rate, greater luminal stenosis reduction following adjunctive angioplasty and a 76.9% freedom from target lesion revascularization at 1 year [31]. The SALVAGE multicenter registry (conducted in nine US centers) also investigated ELA with plain balloon angioplasty for femoropopliteal ISR, but combined with adjunctive heparin-bonded covered stent use. The study was published in 2012 and included 27 patients with IC or CLI and mainly TASC I C and D lesions (81.4%), with a mean lesion length of 20.7 ± 10.3 cm. Although an improvement in all quality-of-life parameters was noted and a 12-month TLR rate of 17.4% was noted, 12-month primary patency was moderate at 48% [32].

In a large retrospective analysis of mixed infrapopliteal atherectomy, Todd et al. investigated 79 BTK atherectomy interventions (33 laser, 13 directional and 33 orbital) alone or with additional balloon angioplasty (68 atherectomy combined with PTA) and compared them with plain balloon angioplasty procedures from a CLI cohort of 418 interventions in total. Procedures were mainly performed for CLI treatment. According to Kaplan–Meier analysis, there was no difference at 12- and 36-month follow-up in primary patency (69, 55% vs. 61, 46%; *p* = 0.158), assisted primary patency (83, 71% vs. 85, 67%; *p* = 0.801), limb salvage (79, 70% vs. 81, 77%; *p* = 0.485) and survival (77, 56% vs. 80, 50%; *p* = 0.944) between balloon angioplasty and atherectomy-assisted group [33].

## Combined Atherectomy and Drug-Coated Balloon (DCB) Treatment

In order to improve patency, the combination of lesion debulking using percutaneous atherectomy and subsequent DCB application has been implemented. Drug-coated balloons are proved to be an effective treatment option that does not require a permanent stent [34–36]. Rates of bailout stenting in of DCB studies range from 4% in the THUNDER (Local Taxane with Short Exposure for Reduction of Restenosis in Distal Arteries) study to 12.3% in the Italian Registry and 21% in the PACIFIER study [34]. One small Italian registry trial was recently published with results from a series of 30 patients with severely calcified SFA lesions. Mean lesion length treated was 115 ± 35 cm and 13% were occlusions. The authors performed intravascular ultrasound-guided directional atherectomy under filter protection using the TurboHawk system, followed by application of a drug-eluting balloon (In.PACT ADMIRAL, Medtronic, USA). The authors reported a very promising 1-year primary patency of 90%, which needs further validation in larger trials. No procedure-related adverse events were noted, and bailout stenting was necessary in 6.5% [37]. Primary angiographic patency was 94.1% when more plaque was removed with directional atherectomy (<30% residual stenosis was achieved) compared to 68.8% patency when less plaque was removed (>30% residual stenosis) before treatment with the DCB [37].

A confirmatory comparative study called the DEFINITIVE AR trial (Atherectomy Followed by a Drug Coated Balloon to Treat Peripheral Arterial Disease; ClinicalTrials.gov Identifier: NCT01366482) has been recently completed and results were presented. This was a multicenter, randomized, controlled trial sponsored by Medtronic, comparing upfront atherectomy with the TurboHawk™ or SilverHawk^®^ plaque excision systems followed by drug-eluting balloon angioplasty versus a drug-eluting balloon (Cotavance™ Drug-Eluting Balloon), as a single approach, in patients with Rutherford 2 to 4 disease due to 7- to 15-cm superficial femoral and/or popliteal lesions. Patients were randomized 1:1 to either directional atherectomy plus DCB group under filter protection (DAART; *n* = 48) or to the paclitaxel-coated balloon alone (*n* = 54). Results were recently presented at the 2015 Charing Cross Symposium by Zeller T. Significantly lower flow-limiting dissection rate was noted in the DAART arm (2 vs. 19%, *p* = 0.01), and the need for bailout stent was only 4.1%. One-year restenosis rate by DUS (PSVR ≤ 2.4, without TLR) and evaluated by an independent core laboratory was 93.4% for the DAART arm and 89.6% for the DCB arm (nonsignificant *p* > 0.05). Angiographic patency (≤50% stenosis and without TLR) again assessed by independent core laboratory was 82.4% in the DAART arm and 71.8% in the DCB arm. DEFINITIVE AR provided higher level of evidence regarding the possible additional benefit of performing debulking atherectomy prior to the use of a drug-eluting balloon. The investigators concluded that the DEFINITIVE AR resulted by trend in potentially better outcomes in challenging lesion subsets such as severely calcified ones, ≥10 cm lesions and CTOs. However, a sufficiently powered study to confirm or refute this potential benefit is still missing. Following this initial experience, Medtronic has launched the REALITY study, a multicenter, prospective, single-arm observational angiographic and duplex ultrasound core laboratory-adjudicated study that will enroll 250 patients at up to 20 US centers to evaluate adjunctive use of directional atherectomy and DCB treatment in patients with symptomatic PAD in long, calcified SFA and/or popliteal artery lesions.

## Discussion

Percutaneous atherectomy offers the capability of minimally invasive atheroma removal or debulking, and vascular specialists are today given the opportunity to choose between a variety of modern and efficient atherectomy devices, based on patient and lesion characteristics. According to currently available data, atherectomy can be effectively and safely used in both femoropopliteal and infrapopliteal diseases. It empirically seems to significantly decrease the need for stenting facilitating future endovascular or open surgical revascularization options and minimizing the risk of occlusion in anatomically “hostile” arterial segments such as flexion points (leaving nothing behind concept). Results from the currently available randomized studies and largest prospective registries are summarized in Table 3.

**Table 3:** Peripheral atherectomy studies (randomized controlled and registries)

Study	Design	Treatment	Patients and lesions	Bailout stent	Immediate outcomes	Clinical outcomes
Shammas et al. [13].	RCT	SilverHawk versus angioplasty	46 IC and 12 CLI femoropopliteal	27.6 versus 62.1% (*p* = 0.017)	Embolization: 64.7 versus 0.0% (*p* < 0.001)	1-year TLR: 11.1 versus 16.7%
DEFINITIVE LE [19]	Multicenter registry	SilverHawk	598 IC 201 CLI 655 femoropopliteal 145 infrapopliteal	3.2%	Embolization: 3.8% Perforation: 5.3%	1-year patency: 78% Amputation: 5%
DEFINITIVE-CA [22]	Multicenter registry	SilverHawk under filter protection	133 patients 168 calcified femoropopliteal	<50% residual stenosis in 92% cases	30-day MAE: 6.9%	N/A
DEFINITIVE AR	RCT	Hawk + DCB versus DCB alone	102 femoropopliteal	Dissection: 2 versus 19%	N/A	1-year patency: 82.4 versus 71.8%
TALON [18]	Multicenter registry	SilverHawk	601 IC + CLI (748 limbs) Femoropopliteal and infrapopliteal	6.3%	Success 97.6%	1-year TLR: 20%
Zeller et al. [23]	Registry	SilverHawk	84 patients IC + CLI (100 limbs)	N/A	Success 100%	1-year patency: 84% de novo 54% restenotic
VISION-IDE	Registry	OCT-guided Pantheris	130 patients 130 lesions	4.0% stenting	N/A	N/A
OASIS [27]	Multicenter registry	Orbital atherectomy	124 patients 201 stenoses Infrapopliteal	2.5% stenting	30-day MAE: 3.2%	6-month improvement 78.2%
COMPLIANCE 360 [15]	RCT	Orbital versus angioplasty	50 patients 65 lesions Femoropopliteal	5.3 versus 77.8% (*p* < 0.0001)	N/A	1-year TLR: 18.8 versus 21.7% (*p* = 0.99)
CALCIUM 360 [14]	RCT	Orbital versus angioplasty	50 CLI Infrapopliteal vessels	6.9 versus 14.3% (*p* = 0.44)	Success: 93.1 versus 82.4% (*p* = 0.27)	1-year TVR: 93.3 versus 80.0% (*p* = 0.14)
PATHWAY [24]	Multicenter registry	Pathway (Jetstream)	172 IC + CLI Femoropopliteal and Infrapopliteal	7%	Success: 99% 30-day MAE: 1%	1-year TLR: 26% Patency: 61.8%
EXCITE-ISR [16]	RCT	Excimer laser versus angioplasty	250 IC + CLI In-stent restenosis	4.1%	30-day MAE: 5.8 versus 20.5% (*p* < 0.0001)	6-month TLR: 26.5 versus 48.2% (*p* < 0.005)
LACI [28]	Multicenter registry	Excimer laser	145 patients 155 limbs Femoropopliteal and infrapopliteal	45%	Success 86% (<50% residual stenosis)	6-month limb salvage: 93%
CELLO [31]	Multicenter registry	Excimer laser	65 IC patients Femoropopliteal	23.3%	N/A	1-year Patency: 54% TLR: 23.1%

Notably, despite the advantages of endovascular plaque removal, moderate barotrauma and absence of metallic mesh which has been known to induce inflammation, percutaneous atherectomy has not significantly reduced restenosis rates compared to standard endovascular therapy. Nonetheless, this could be attributed to study design, as the role of atherectomy in terms of patient and lesion selection remains to be determined. Specifically, each type of atherectomy is characterized by specific advantages and disadvantages which might influence immediate technical success and primary patency following treatment of different lesions with diverse morphology such as severe calcified plaque, eccentric lesions and CTOs.

Another major disadvantage of percutaneous atherectomy devices, including those with active debris removal function, is the risk of distal embolization, and therefore, distal filter protection is mandatory. In the PROTECT (Preventing Lower Extremity. Distal Embolization Using Embolic Filter Protection) registry which investigated distal embolization events using peripheral filters in 40 patients, clinically significant macrodebris (diameter > 2 mm) was evident in 90.9% of the atherectomy patients versus 27.6% of the angioplasty/stenting patients [38]. Considering the routine use of larger sheath sizes to accommodate transcatheter atherectomy, increased procedural time and similar patency rates, it is obvious that there is not enough evidence to recommend percutaneous atherectomy as primary means of treatment over balloon angioplasty and/or stenting yet [17]. However, the unique aspects of this endovascular treatment modality may provide a net advantage over standard endovascular treatment in selected patients with increased lesion complexity (heavy calcium, longer lesions, chronic total occlusions) and no stent zones like the common femoral and popliteal anatomy. Formal evidence about transcatheter atherectomy in the subintimal space is missing, and there is a relative contraindication to apply most atherectomy devices in the subintimal plane because of the presumed risk of vessel perforation due to an inadvertent adventitial cut. The latter may be more relevant to directional plaque removal; however, the authors advise caution in general. In theory, image-guided devices (e.g., OCT-guided Pantheris) may be beneficial in recognizing the vessel boundaries and performing volumetric plaque removal even in complex subintimal channels.

A very interesting concept is “lesion preparation” and the “leaving nothing behind” approach, where atherectomy is employed for plaque debulking and modification prior to drug-coated balloon (DCB) angioplasty so as to maximize acute luminal gain, remove/remodel the calcium barrier, facilitate drug diffusion and minimize the need for stenting. In that way, vessel wall is cleared from atheromatic plaque, there is less need for high-pressure angioplasty, and therefore, low-pressure DCB angioplasty could suffice while at the same time local drug delivery is optimized with more uniform and deeper drug transfer for inhibition of restenosis. Moreover, low-pressure balloon angioplasty could limit vessel wall barotraumas and further limit inflammatory response of the arterial wall. This interesting concept of combined atherectomy and DCB treatment remains to be better determined by well-designed randomized controlled trials (RCT), as solid data demonstrating its superiority over standard endovascular treatment are currently insufficient [5, 8].

## Future Perspectives

An even more interesting treatment combination would be atheroma debulking using percutaneous atherectomy followed by bioabsorbable drug-eluting stent deployment. In this way, all possible advantages of endovascular technologies, such as transcatheter plaque excision, optimal lesion preparation to allow placement of a bioresorbable scaffold, maximal luminal gain without elastic recoil, long-lasting drug delivery for inhibition of neointimal hyperplasia, as well as no permanent metal implant, will be combined in order to provide the optimal treatment effect for PAD patients. Further technological developments that will minimize the risk of distal embolization, reduce device profile and accelerate procedural time could also be key elements in order to establish percutaneous atherectomy as the first-line endovascular PAD treatment option with superior patency outcomes and no permanent metal stents.

In conclusion, percutaneous transcatheter atherectomy can achieve significant plaque removal and downstage the anatomical complexity of PAD. Current evidence indicates that femoropopliteal and infrapopliteal percutaneous atherectomy may achieve a high technical success with significantly lower bailout stenting rates. On the other hand, there is absence of robust evidence that long-term patency results are improved by the application of transcatheter atherectomy alone. Therefore, high-quality data from studies investigating novel hybrid treatment strategies, for example combination of atherectomy and drug-eluting technologies, are necessary.

## Abbreviations

PAD: Peripheral arterial disease
FR: French size of catheters
IFU: Instructions for use
CTO: Chronic total occlusions
FDA: Food and Drug Administration
OCT: Optical coherence tomography
RCT: Randomized controlled trial
LASER: Light Amplification by Stimulated Emission of Radiation
ELA: Excimer laser angioplasty
TLR: Target lesion revascularization
TVR: Target vessel revascularization
CLI: Critical limb ischemia
IC: Intermittent claudication
BTK: Below-the-knee
DCB: Drug-coated balloons
HR: Hazard ratio
DUS: Duplex ultrasound
